# Genome Sequence of Lassa Virus Isolated from the First Domestically Acquired Case in Germany

**DOI:** 10.1128/genomeA.00938-16

**Published:** 2016-09-22

**Authors:** Svenja Wolff, Tilman Schultze, Sarah Katharina Fehling, Jan Philipp Mengel, Gerrit Kann, Timo Wolf, Markus Eickmann, Stephan Becker, Torsten Hain, Thomas Strecker

**Affiliations:** a Institute of Virology, Philipps University Marburg, Marburg, Germany; b German Center for Infection Research (DZIF), Partner Site Giessen-Marburg-Langen, Germany; c Institute for Medical Microbiology, Justus-Liebig University Giessen, Giessen, Germany; d Department of Medicine, Infectious Diseases Unit, University Hospital Frankfurt, Frankfurt am Main, Germany

## Abstract

Lassa virus (LASV) is a zoonotic, hemorrhagic fever-causing virus endemic in West Africa, for which no approved vaccines or specific treatment options exist. Here, we report the genome sequence of LASV isolated from the first case of acquired Lassa fever disease outside of Africa.

## GENOME ANNOUNCEMENT

Lassa virus (LASV), a member of the *Arenaviridae* family (genus *Mammarenavirus*), is the causative agent of human Lassa fever disease, an acute viral hemorrhagic fever endemic in West Africa. Major outbreaks are most frequently observed in Guinea, Liberia, Nigeria, and Sierra Leone; however, isolated cases and serological evidence of LASV infections have also been reported in Benin, Burkina Faso, Ghana, Ivory Coast, Mali, and Togo, indicating a large geographical area of LASV endemicity in West Africa (1, 2). The wide geographic spread of LASV is probably linked to its natural rodent reservoirs. The primary reservoir is the multimammate rat *Mastomys natalensis*, though LASV has also been isolated from other rodent species (3, 4). While most human infections result from zoonotic transmission, human-to-human transmission can occur, particularly during nosocomial outbreaks (5).

In February and March 2016, Germany reported two LASV cases. The index case involved a U.S. citizen who worked as a healthcare provider in the northern part of Togo. He was medically evacuated to Cologne on 25 February 2016, but died one day later with the cause of death being unknown at that time. Lassa fever disease was diagnosed during a postmortem examination (6). The second case was a German funeral home worker who handled the deceased body, representing the first person who contracted LASV outside of Africa (7).

We have determined the genome sequence of the bisegmented ambisense RNA genome of LASV, isolated from the secondary case. To this end, an LASV-positive blood sample collected from the patient on the day of admission to the Frankfurt University Hospital High-Level Isolation Unit was used to inoculate Vero E6 cells. Cell culture supernatant from infected cells collected at 5 dpi was freed from cellular debris and then subjected to ultracentrifugation through a 20% sucrose cushion as previously described (8). RNA was extracted from pelleted virions using an RNeasy minikit (Qiagen, Germany) (sequences were derived from p1 stocks). A cDNA library for sequencing was prepared from the isolated RNA, applying Illumina’s TruSeq total RNA kit, including rRNA depletion, as described previously (9). The library was sequenced on the Illumina MiSeq platform using v3 chemistry and paired-end sequencing (2 × 300 cycles). Sequence reads were aligned to LASV L- and S-segment references (KU961971.1 and KU961972.1). When only reads mapping in intact paired-end orientation were considered, we obtained average coverages of 165× (median 145×) for the L- segment and 225× (median 185×) for the S-segment, respectively. Poorly covered regions, such as the noncoding intergenic region of the L-segment, as well as detected single-nucleotide variants, were reevaluated using conventional Sanger sequencing.

Our sequence data will help to inform diagnostic laboratories and provide a source of information for evolutionary and epidemiological analysis of LASV circulating in Togo.

### Accession number(s).

The genome sequences of the Lassa mammarenavirus isolate Alzey have been deposited at the European Nucleotide Archive under the accession numbers LT601601 and LT601602.
